# Ocular Drug Delivery Barriers—Role of Nanocarriers in the Treatment of Anterior Segment Ocular Diseases

**DOI:** 10.3390/pharmaceutics10010028

**Published:** 2018-02-27

**Authors:** Rinda Devi Bachu, Pallabitha Chowdhury, Zahraa H. F. Al-Saedi, Pradeep K. Karla, Sai H. S. Boddu

**Affiliations:** 1 College of Pharmacy and Pharmaceutical Sciences, The University of Toledo Health Science Campus, Toledo, OH 43614, USA; RindaDevi.Bachu@rockets.utoledo.edu (R.D.B.); Pallabita.Chowdhury@rockets.utoledo.edu (P.C.); Zahraa.AlSaedi@rockets.utoledo.edu (Z.H.F.A.-S.); 2 Department of Pharmaceutical Sciences, College of Pharmacy, Howard University, 2300 4th St. NW, Washington, DC 20059, USA

**Keywords:** anterior segment, novel drug delivery systems, polymeric nanocarriers, disposition, toxicity

## Abstract

Ocular drug delivery is challenging due to the presence of anatomical and physiological barriers. These barriers can affect drug entry into the eye following multiple routes of administration (e.g., topical, systemic, and injectable). Topical administration in the form of eye drops is preferred for treating anterior segment diseases, as it is convenient and provides local delivery of drugs. Major concerns with topical delivery include poor drug absorption and low bioavailability. To improve the bioavailability of topically administered drugs, novel drug delivery systems are being investigated. Nanocarrier delivery systems demonstrate enhanced drug permeation and prolonged drug release. This review provides an overview of ocular barriers to anterior segment delivery, along with ways to overcome these barriers using nanocarrier systems. The disposition of nanocarriers following topical administration, their safety, toxicity and clinical trials involving nanocarrier systems are also discussed.

## 1. Introduction

**Figure 1:**
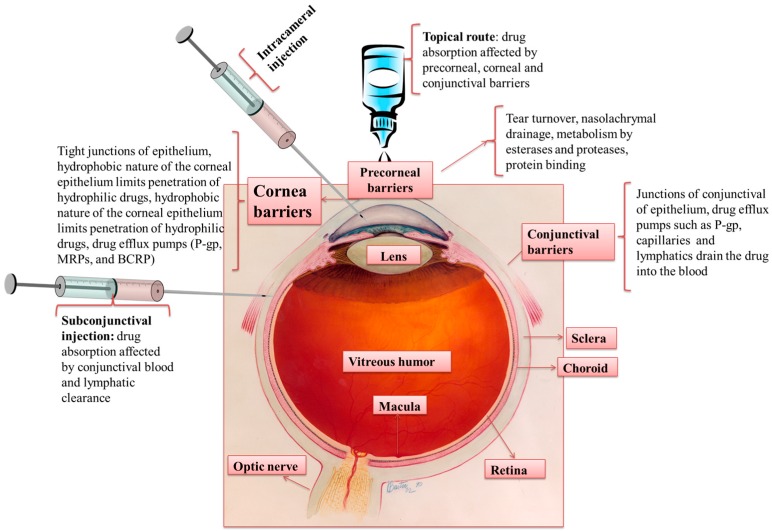
Routes of administration for anterior segment drug delivery, ocular tissue barriers and clearance mechanisms that prevent drug absorption into the eye. Modified from: Credit: National Eye Institute, National Institutes of Health (Ref#: NEA04).

The human eye is a complex organ with intricate anatomical and physiological barriers. The anterior segment of the eye consists of the cornea, conjunctiva, aqueous humor, iris, ciliary body, and lens. The posterior segment mainly consists of the vitreous humor, retina, choroid, and optic nerve (Figure 1) [1,2]. Common diseases affecting the anterior segment of the eye are dry eye syndrome, glaucoma, allergic conjunctivitis, anterior uveitis, and cataract. Prominent diseases affecting the posterior segment of the eye include age-related macular degeneration (AMD), diabetic retinopathy macular edema (DME), proliferative vitreoretinopathy (PVR), posterior uveitis, and cytomegalovirus (CMV) [1]. The global population includes 258 million visually impaired people, with 39 million among them being completely blind [3]. These concerning statistics validate an increased need for exploring improved drug delivery strategies for ocular therapies.

Drug delivery to the anterior segment of the eye via the topical route typically involves the conventional dosage forms, such as solutions (62.4%), suspensions (8.7%), and ointments (17.4%), which compose an estimated 90% of marketed ophthalmic formulations. However, topical drug administration demonstrates poor ocular bioavailability (<5%) due to lacrimal secretions that lead to poor retention time and decreased permeability across the corneal epithelium. Tear turnover from lacrimal secretions contributes to a majority of the drug loss. A healthy eye with a tear volume of ~7–9 µL has a turnover rate of 0.5–2.2 µL/min [4]. During topical administration, the average volume of major formulations is ~35–56 µL, and excess volume drains via the nasolacrimal duct into systemic circulation. In addition, conjunctival blood circulation affects the topical drug absorption. All of the barriers combined result in a drug loss of ~95% from topical administration. The rest of the drug encounters the corneal epithelial barrier. The significant role of the cornea as a barrier is discussed in detail in subsequent sections.

## 2. Anterior Segment Drug Delivery Barriers

### 2.1. Epithelial Tight Junction (ZO)

The corneal epithelium forms the primary barrier to drug absorption via topical administration. The stratified corneal epithelium consists of a basal layer of columnar cells, two to three layers of wing cells and one or two outer layers of squamous cells [5]. Superficial cells are surrounded by the intercellular tight junctions (*zonula occludens*). The tight junctions act as barriers for permeation of drug molecules via the paracellular route. Tight junctions are composed of anastomotic strands that confer resistance to the paracellular drug absorption [6]. There are four tight junction proteins, ZO-1, cingulin, ZO-2 [7], and occluden [8], with occluden being the most important. Extracellular and intracellular calcium levels in tight junctions influence the permeability [9]. If tight junction membrane integrity is disrupted or extracellular calcium ions are removed by EDTA, drug permeability increases throughout the tight junctions [10,11]. The pores of the corneal epithelium are negatively charged at physiological pH, hence negatively charged molecules permeate slowly compared to positively charged molecules [12]. Also, the cellular calcium levels and actin filaments present on the cytoskeleton play an important role in maintaining the integrity of tight junctions [6,9,10].

### 2.2. Reflex Blinking

A normal eyedropper delivers 25–56 µL of the topical formulation with an average volume of 39 µL. However, an eye can transiently hold up to 30 µL, and the rest is lost either by nasolacrimal drainage or reflex blinking (5–7 blinks/min), significantly decreasing the overall drug available for therapeutic action [13].

### 2.3. Metabolism in Ocular Tissues

Drugs containing aromatic hydrocarbons are metabolized in the pigmented epithelium and ciliary body to their corresponding epoxides and phenols, or further metabolized by other enzymes present in the eye [14]. Hayakawa et al. demonstrated that poor absorption of peptide drugs and insulin is due to extensive metabolism during conjunctival permeation in albino rabbits [15]. Schoenwald et al. demonstrated that clearance via aqueous humor turnover is significantly lower when compared to the rest of the clearance pathways, indicating that a majority of the drugs are eliminated via metabolic pathways [16,17].

### 2.4. Tear Turnover

A significant impediment to topical ocular delivery is tear turnover. Following topical administration, an increase in the volume of cul-de-sac occurs that leads to reflex blinking and increased tear secretion, eventually resulting in rapid drug loss from the precorneal area [18]. Loss of the solution occurs due to tear turnover and nasolacrimal drainage until the tear volume in the conjunctiva cul-de-sac returns to a normal range (7–9 µL) [19]. The initial first order drainage rate of eye drops from the ocular surface is 1.2 µL/min in humans [17,20] and 0.5–0.7 µL/min in rabbits [21].

### 2.5. Nasolacrimal Drainage

As mentioned above, a majority of the instilled drug is lost due to tear turnover or nasolacrimal drainage. About 95% of the dose administered is eliminated systemically via the conjunctiva and nasolacrimal duct [22]. The lacrimal drainage system in human adults serves as a conduit for tear flow from the eye to the nasal cavity. The pathway consists of the puncta, canaliculi, lacrimal sac, and nasolacrimal duct. Histologically, the walls of the lacrimal sac and the nasolacrimal duct are vascularized, and hence are potential sites for systemic drug absorption. After topical application, the eye drop solution initially mixes with lacrimal fluid. The contact time of the drug with ocular tissues is approximately 1–2 min due to the constant production of lacrimal fluid. Approximately half of the drug flows into the upper canaliculus and the rest into the lower canaliculus of the lacrimal sac. The flow further opens into the nasolacrimal duct, and, from there, drains into the nose [23]. A few factors that determine the topically applied drug concentration are the volume of the instilled drug solution, reflex blinking by the patient and the patient’s age. Larger instilled volumes easily pass into the nose from the nasolacrimal sac [24], and smaller volumes are easily eliminated from the lacrimal sac [25]. The loss of drugs from the nasolacrimal duct via transconjunctival absorption or transnasal absorption is unwanted because of direct exposure to systemic circulation [26].

### 2.6. Efflux Pumps

The efflux proteins are located either on the apical or basolateral cell membranes. These proteins either restrict or enhance the drug absorption, depending on their cellular localization [27]. The ATP-binding cassette, commonly known ABC proteins, are a superfamily of proteins that are encoded by an MDR1 gene responsible for the efflux of various substrates across the plasma membrane and cytoplasm into the extracellular fluid. There are primarily two major efflux pumps that are responsible for drug resistance: (a) P-glycoprotein, which restricts entry of amphipathic compounds, both in normal and cancer tissue, and (b) multidrug resistant protein (MRP) (ABCC1), which is known to efflux organic anions and conjugated compounds [27,28].

P-glycoprotein 1 (P-gp), also known as MDR1 or ABCB1, is a ~170 kDa ATP dependent efflux pump. It is located on the apical surface of polarized cells [29] and is responsible for decreasing drug accumulation in multidrug-resistant cells. Further, it confers resistance to the absorption of anticancer drugs by tumor cells. P-gp was shown to be present on the ocular conjunctival epithelial cells [30], ciliary non-pigmented epithelium [31], human and rabbit cornea [32], iris and ciliary muscle cells, and retinal capillary endothelial cells [33]. P-gp has been detected at the mRNA level in the human cornea, rabbit corneal epithelium, and primary cultures of rabbit corneal epithelial cells [32]. According to Constable et al. the presence of P-gp on three human RPE cells (ARPE19, D407, and h1RPE) have been studied. It was demonstrated that only D407 cells express P-gp and can be utilized for in vitro drug transport studies without any modifications [34].

MRP is a ~190 kDa membrane-bound efflux protein encoded by the ABCC1 gene. It is generally found on the basolateral surface of the intestine, hepatocytes, and kidney cells [35,36]. It acts as an organic anionic transporter with glutathione, cysteinyl leukotrienes, glucuronides, sulfate conjugates and bile salts [37]. MRP expression has been detected in the human corneal epithelium at the RNA level [38]. MRP5 was expressed at a higher level than MDR1, MRP1–MRP4, MRP6, and BCRP [27]. Chen et al. investigated the expression sites of various efflux transporters in different ocular tissues. The study reported that in human cornea efflux transporters including MRP1–4, MRP6 were localized in the basal layer of corneal epithelium, whereas MRP7 and MDR1 were expressed in the entire corneal epithelium. In human conjunctiva, MRP2–4, MRP6, MDR1, and BCRP were expressed in basal cell layer while MRP1, MRP7 were detected in the entire conjunctival epithelium. In human iris ciliary body, MRP1–2, MDR1 were detected in stromal cells [39]. Zhang et al. studied drug transporter and cytochrome P450 mRNA expression in ocular drug disposition. They concluded that both BCRP and MRP2 have very low expression levels in the human cornea, while MRP1 was moderate and MRP3 had low expression levels in the human cornea. Thus, designing drugs that could efficiently evade MRP1 efflux can play an important role in enhancing the ocular absorption [40].

## 3. Nanocarriers for Anterior Segment Drug Delivery

Despite extensive research efforts, drug delivery to the eye remains a challenge. The anatomical position of the eye confers a unique advantage for site-specific drug delivery and non-invasive clinical assessment of a disease state. For optimal therapeutic activity, drug molecules should circumvent the protective physiological barriers without causing permanent tissue damage. Anterior segment drug delivery comprises the conventional dosage forms, such as solutions, suspensions, ointments and novel dosage forms, such as liposomes, nanoparticles, and implants [41,42]. However, >90% of the marketed formulations are conventional dosage forms, with limited bioavailability due to precorneal clearance and less duration of action, thus requiring frequent administration [43]. Major research is directed towards the development of sustained release nanocarrier systems with higher precorneal retention. Such systems can improve the ocular bioavailability of drugs and provide high patient compliance. For example, patient adherence to eye drops plays a key role in the management of glaucoma and is frequently low (<50%) [44]. Administration of drugs to the eye by means of a droptainer bottle is challenging in elderly patients, due to the lack of physical acuity and inability to aim adequately [45]. The adherence to antiglaucoma therapy using eye drops deteriorates with age [46]. Nanocarriers, such as liposomes, micelles, microemulsions, biodegradable nanoparticles, nanosponges, punctal plugs, and dendrimers hydrogels have been investigated as carriers for antiglaucoma drugs for their ability to deliver drugs in a sustained manner [47]. Further, precorneal retention of drugs loaded into nanocarriers has been improved by coating them with mucoadhesive polymers such as polyethylene glycol (PEG), chitosan and hyaluronic acid, and by dispersing nanocarriers in stimuli-responsive hydrogel, such as pH-, thermo-, and ion-sensitive hydrogels. Nanocarriers were found to effective in the prevention and treatment of cataract, where the nanodrug reached higher lens concentrations; while, the free drug was washed away by tears [48]. More recent research efforts are focused on identifying enhanced drug permeability across the cornea via nanocarrier-mediated tight junction reorganization effect [49]. The most widely employed nanoformulations in treating anterior segment diseases will be discussed in detail in the subsequent sections.

### 3.1. Microemulsions

**Figure 2:**
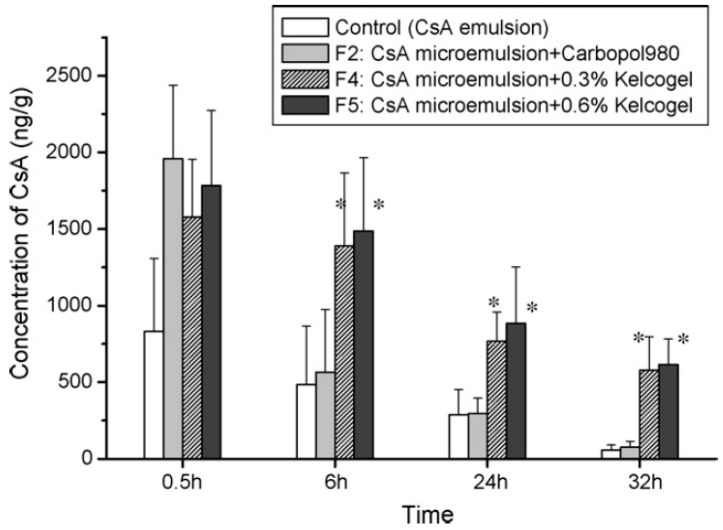
Concentration of cyclosporine A (CsA) in the cornea after the instillation of formulations (* *p* < 0.05) Reprinted from [59]. Copyright (2018), with permission from Elsevier.

A microemulsion is a dispersion of water and oil stabilized by surfactants or co-surfactants to reduce the interfacial tension. Microemulsions are clear in appearance and thermodynamically stable with a small droplet size (~100 nm). Microemulsion formulations are shown to increase the solubility of drugs. An oil-in-water type of microemulsion in the presence of surfactant and co-surfactant is able to increase corneal membrane permeability [50]. Increased permeability and sustained release of drugs makes microemulsions an attractive vehicle for ophthalmic drugs. Microemulsification improved solubility of poorly soluble drugs, such as indomethacin and chloramphenicol [51]. Microemulsions have low surface tension and high spreading coefficient, allowing for the drug to spread and mix well with the precorneal fluid. This improves the corneal contact time of drugs [52]. They can be sterilized by filtration for formulation as eye drops. Many studies have reported the occurrence of electrostatic attraction between the emulsified cationic droplets and anionic cellular charges of ocular tissues. Incorporation of a positively charged lipid might therefore increase the binding of cationic droplets to the negatively charged corneal surface [53]. Microemulsion formulations of the ocular drugs, indomethacin, delta-8-tetrahydrocannabinol, pilocarpine, and timolol were tested extensively [54]. In vivo rabbit studies using microemulsions demonstrated a sustained release effect and improved bioavailability [55]. The pilocarpine microemulsion demonstrated increased absorption and reduced dosing frequency to twice a day, as compared to four times a day with conventional eye drops [56]. Pilocarpine microemulsion systems exhibited different morphological forms (crystalline liquid and emulsion) with changes in aqueous content. This altered rheological behavior contributed to higher viscosity and longer retention of the formulation on the corneal surface [57]. The moxifloxacin-loaded water-in-oil microemulsion demonstrated sustained drug release with higher in vivo antimicrobial activity as compared to the conventional solution [58]. Gan et al. developed a cyclosporine-loaded microemulsion with in situ gelling capacity. The developed formulation demonstrated prolonged residence time, with three times higher AUC, compared to the conventional emulsion. Further, the formulation resulted in a sustained cyclosporine delivery for 32 h, preventing the corneal allograft rejection (Figure 2) [59].

Despite advantages, a narrow range of surfactants and oils that are non-toxic and biocompatible limits the success of microemulsions in ocular drug delivery [54]. A review by Hedge et al. provided detailed information on microemulsions for ocular drug delivery [60].

### 3.2. Nanosuspensions

Nanosuspensions are sub-micron colloidal dispersions of poorly water-soluble drugs in a dispersion medium stabilized by surfactants or polymers. These formulations usually consist of a colloidal carrier, such as a polymeric resin, which is inert in nature, for enhancing drug solubility and bioavailability. Unlike microemulsions, they are non-irritant and are regarded a desirable ocular drug delivery vehicle [61]. The inert carriers employed in nanosuspensions are non-irritating to the cornea, iris, and conjunctiva [62]. Nanosuspensions increase the precorneal residence time and enhance solubility and ocular bioavailability of drugs. Glucocorticoids, such as dexamethasone, prednisolone, and hydrocortisone are widely used in treating anterior segment inflammatory diseases [61]. Repeated administration of glucocorticoid doses was clinically shown to induce cataract formation and cause damage to the optic nerve. Nanosuspension formulations of corticosteroids resulted in sustained drug release and increased ocular bioavailability [63]. Flurbiprofen helps to decrease post-surgical edema after intra-ocular surgery, and flurbiprofen-loaded polymeric nanosuspension has been shown to prevent myosis during extracapsular cataract surgery [64,65]. The positive charge on nanoparticles increases adherence with the negatively charged corneal surface [66]. A lomefloxacin HCl-loaded nanosuspension demonstrated a three-fold increase in drug permeation across bovine corneas as compared to the parent drug solution. Further, a ~3.5 fold decrease in minimum inhibitory concentration (MIC) against gram-negative bacteria was observed [67]. A similar study using moxifloxacin-loaded nanosuspension showed sustained drug release and comparable in vitro corneal permeability to that of Moxicip^®^, a marketed product.

**Figure 3:**
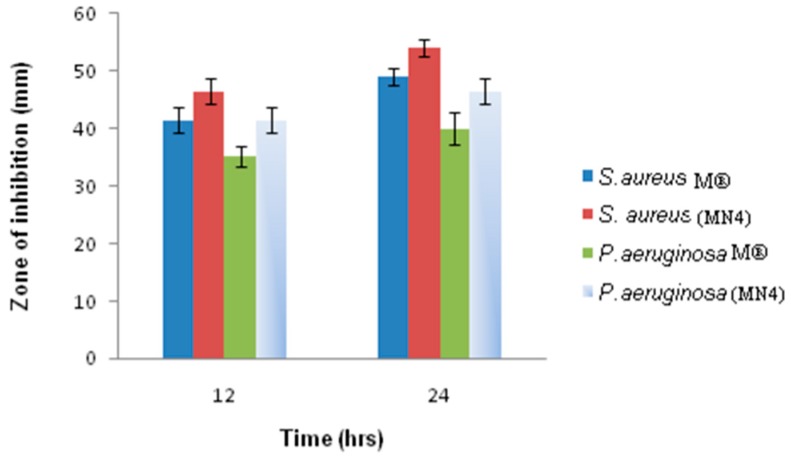
Anti-bacterial activity of optimized moxifloxacin-loaded nanosuspension (MN4) vs. marketed eye drops (M^®^) [68].

Also, the optimized nanosuspension demonstrated higher anti-bacterial activity against *S. aureus* and *P. aeruginosa* (Figure 3) when compared to conventional eye drops [68]. In vivo pharmacokinetic studies showed that diclofenac/MPEG-PCL-CS (chitosan grafted methoxy poly(ethylene glycol)-poly(ε-caprolactone)) nanosuspension enhanced the pre-corneal retention time, permeation and bioavailability of diclofenac compared to the marketed eye drops [69]. These studies demonstrated that nanosuspensions are an attractive alternative to conventional eye drops for ocular drug delivery.

### 3.3. Liposomes

Liposomes are lipid vesicles composed of one or more phospholipid bilayers with a central aqueous compartment and are 0.025–10 µm in diameter. They are capable of incorporating both hydrophilic and lipophilic drugs due to the presence of a central aqueous compartment and lipid layer. Liposomes have a higher degree of biocompatibility than a polymer-based system [70]. Liposomes can adhere to the cornea and are favorable for drugs with low solubility, low partition coefficient, high molecular weight, and poor absorption [71]. The positive charge on liposomes allows for them to bind to the negatively charged mucin coating on the corneal epithelium. A positively charged liposome is shown to enhance the transcorneal flux of penicillin G four-fold, suggesting enhanced corneal permeability [71]. A review of literature shows that liposomal drug delivery is more favorable for lipophilic as compared to hydrophilic drugs. Ganciclovir liposomes demonstrated two to ten times higher drug concentrations in the sclera, cornea, iris, lens, and vitreous humor compared to the ganciclovir solution [72]. C6-ceramide loaded in liposomes was shown to be capable of treating inflammations of the anterior segment of the eye [73].

**Figure 4:**
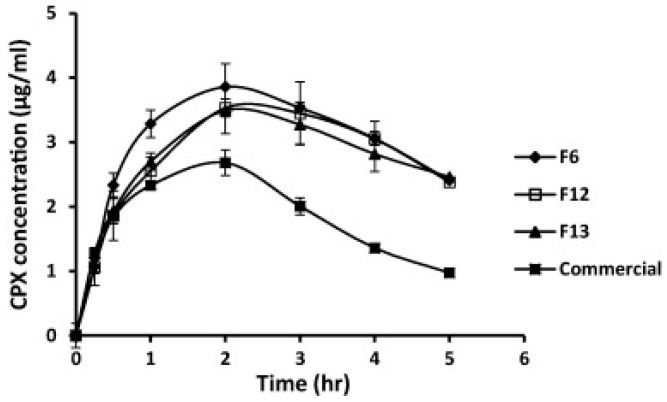
Concentration of ciprofloxacin in rabbit aqueous humor after the instillation of various liposomal formulations (F6, F12, and F13) as well as commercial eye drops. F6, F12, F13 are prepared using varying molar ratios of phosphatidylcholine (PC), Dipalmitoylphosphatidylcholine (DPPC), 1,2-Dimyristoyl-sn-glycero-3-phosphocholine (DMPC), cholesterol (CH), stearylamine (SA) and dioctadecyldimethyl ammonium bromide (DODAB). Reprinted from [74]. Copyright (2018), with permission from Elsevier.

In vivo pharmacokinetic studies with selected ciprofloxacin liposomal formulations F6, F12, and F13 in albino rabbits revealed that prepared liposomes demonstrated enhanced aqueous humor concentrations (Figure 4), and a three-fold higher bioavailability when compared to the commercial eye drops (Ciprocin^®^) [74]. A single dose of latanoprost liposomal formulation significantly lowered intra-ocular-pressure for 90 days in rabbits, compared to a daily topical dose of latanoprost [75,76]. Although liposomes confer advantages similar to eye drops and reduce the dosing frequency, short shelf-life, limited drug-loading capacity, and sterilization issues limit their use [62].

### 3.4. Dendrimers

These formulations are polymeric macromolecules with highly branched star-shaped structures. Dendrimers are nanoconstructs with unique physical and chemical properties such as higher water solubility, encapsulation ability, monodispersity, and surface functionalizable groups. The ability to functionalize surface groups makes them a suitable candidate for delivery of both hydrophilic and lipophilic drugs [77,78]. Earlier bioadhesive polymers, such as poly (acrylic) acid were utilized to improve ocular drug delivery by prolonging contact time for better absorption. However, blurring of vision and formation of a veil in the precorneal area, leading to vision loss, limits the use of polymer [79]. To overcome the limitation, dendrimers consisting of polyamidoamine (PAMAM) with carboxylic and hydroxyl surface groups were introduced. PAMAM was able to increase the number of branches in the dendrimers, leading to the development of higher generation dendrimer (G0, G1, G2, and so on), where G stands for “generation”. PAMAM not only improves drug solubility but also allows for surface conjugation of the targeting ligand and/or drugs. Dendrimers are suitable for ophthalmic drug delivery since they can solubilize lipophilic and hydrophilic drugs in their core and have an exterior region of terminal moieties providing sustained drug release [80,81,82]. Vandamme et al. studied the effect of pilocarpine nitrate and tropicamide utilizing PAMAM dendrimers and found that the bioavailability was improved due to better bioadhesion and sustained drug release [83]. Drug delivery using dendrimers could be further improved by PEGylation of dendrimer surfaces. Drug delivery by dendrimers can be altered by selecting the appropriate surface groups, such as amine, carboxylic, and hydroxyl, or selecting the size or molecular weight of the dendrimers. A detailed review about types, properties of dendrimers and ocular application of dendrimeric delivery systems is described by Yaruz et al. [84].

### 3.5. Niosomes and Discomes

Niosomes are bilayered, nanosized vesicles made up of amphiphilic nonionic surfactants that are biodegradable, biocompatible, and non-immunogenic. They are chemically stable with 10 to 1000 nm in size and are capable of incorporating both hydrophilic and lipophilic drugs [62]. Another novel carrier system is the discome, which is a large structure (12–16 mm) as derived from niosomes by the addition of non-ionic surfactants such as Solulan C24 [85]. Niosomes are preferred carrier systems for ocular drug delivery due to their low toxicity that is associated with the use of nonionic surfactants. Niosomes do not require special handling during preparation and are shown to provide targeted, sustained drug release, and enhanced bioavailability. A niosomal formulation of cyclopentolate delivered the drug independent of pH and significantly improved bioavailability [71].

**Figure 5:**
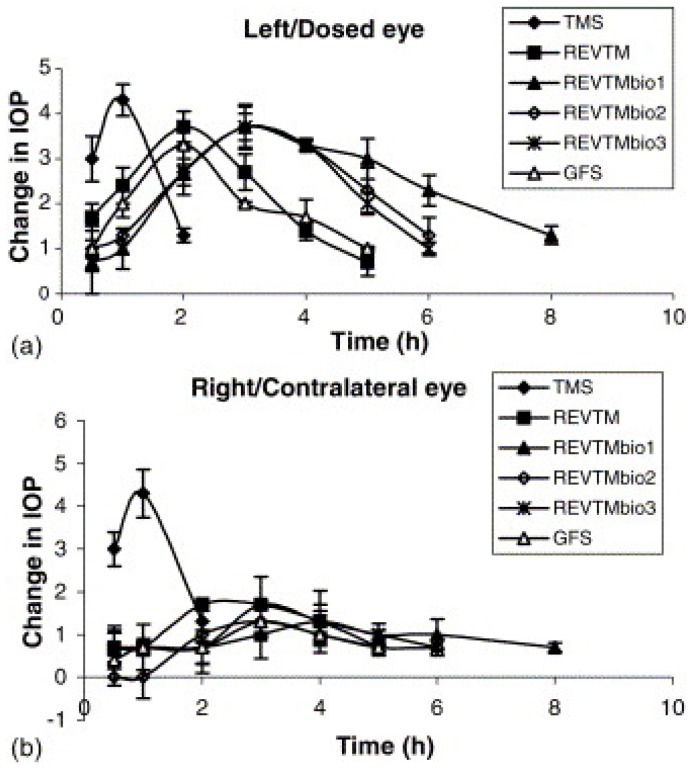
Change in intraocular pressure (IOP) for various formulations in (**a**) dosed eye (**b**) contralateral eye (*n* = 6). (TMS—Timolol solution 0.25%, REVTM—Uncoated niosomes, REVTMbio1-chitosan coated niosomes, REVTMbio2 and 3-carbopol coated niosomes, GFS—Timolet^®^ GFS). Reprinted from [86]. Copyright (2018), with permission from Elsevier.

According to Aggarwal et al. timolol maleate niosomes coated with chitosan and carbopol lowered the intraocular pressure in albino rabbits in a controlled manner for 8 h when compared to a timolol maleate solution (Figure 5) [86]. Novel elastic niosomes (ethoniosomes) were developed for ocular delivery of topical corticosteroids, including prednisolone acetate and prednisolone sodium phosphate. The prepared ethoniosomes showed no ocular irritation, and the bioavailability was 1.75 and 1.54 times higher than the Prednisol^®^ eye drops and Predforte^®^ suspension, respectively. Moreover, the side effect of intraocular pressure (IOP) elevation observed with the commercial products was significantly lower with ethoniosomes [87]. An ex vivo study of transcorneal permeability reveals that niosomes can provide sustained drug delivery and improved corneal permeation [88]. Therefore, niosomes can be considered as a safe option for sustained transcorneal drug delivery. Discomes, when prepared, cause the surfactant to partition into the lipid bilayer, forming a large disc-like structure. Discomes have a longer residence time in the cul-de-sac and less systemic drainage due to their large size [71]. Entrapment efficiency of drugs is also higher with discomes than with niosomes. In vivo bioavailability of drugs in discomes was found to be better than in niosomes, as reported by Vyas et al. [85]. It was found that the entrapment efficiency of naltrexone hydrochloride is five times greater in discomes than in niosomes.

### 3.6. Cubosomes

Cubosomes are self-assembled liquid crystalline particles or nanoparticles [89]. Cubosomes that are loaded with dexamethasone were developed with monoolein and polymer 407, employing an emulsification technique. Ethyl rhodamine B (Rh B) was used to label the nanoparticles, whereas Rh B solutions and Rh B carbopol gel served as controls. It was found that dexamethasone, in cubosomes, increases the AUC by 2.5–3.5 times, relative to Rh B solutions and Rh B carbopol gel. Further, no significant difference in clearance rate was observed for the control groups. This indicates that cubosomes were able to improve the ocular residence time and bioavailability of the drug in ocular tissues.

**Figure 6:**
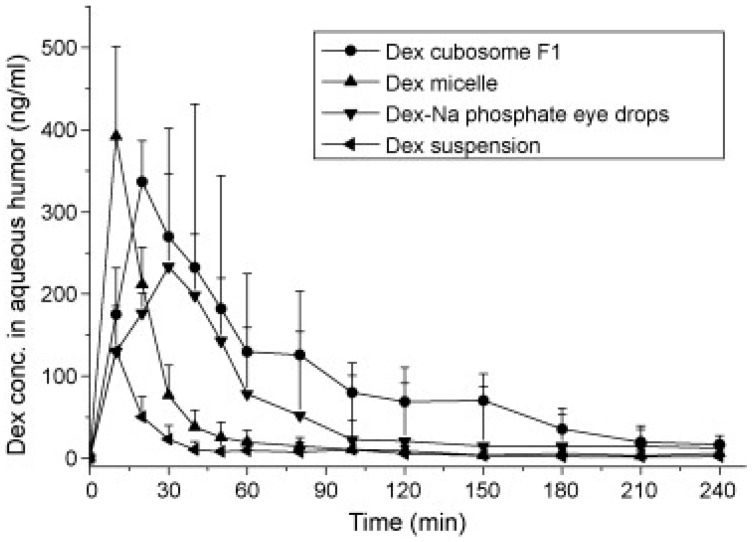
Concentration-time profile of Dexamethasone (Dex) in the aqueous humor of rabbit after the instillation of various formulations. Reprinted from [89]. Copyright (2018), with permission from Elsevier.

An in vivo microdialysis study revealed that dexamethasone in cubosomes increased the drug concentration in the aqueous humor by 1.8-fold, when compared to dexamethasone eye drops, and by eight-fold when compared to a dexamethasone suspension (Figure 6). This study concluded that cubosomes can be a good alternative to conventional solutions [89].

### 3.7. Nanomicelles

Nanomicelles are colloidal, structured carrier systems that range from 5 to 200 nm in size. They are made up of amphiphilic surfactant molecules that may be anionic, cationic or zwitterionic in nature, or diblock polymers [70]. Micelles could be spherical, cylindrical, or star-shaped, depending on the molecular weight of the core and corona forming blocks [90]. These amphiphilic molecules orient themselves to form normal or reverse micelles. Normal micelles are formed when the hydrophobic portion forms a cluster in the core and the hydrophilic part aligns outwards, increasing the contact with water. Similarly, when an opposite alignment occurs, the clustered aggregates are called reverse micelles. Normal micelles are employed to encapsulate, solubilize, and deliver hydrophobic drugs, whereas reverse nanomicelles are utilized to encapsulate and deliver hydrophilic drugs [91]. Nanomicelles are regarded as safe alternatives for ocular drug delivery because of their ability to solubilize less water-soluble drugs in the hydrophobic core and form a clear aqueous formulation. The primary advantages of nanomicelles include ability to formulate hydrophobic drugs into a clear aqueous solution, high water solubility, monodispersity, ability to form nanosize constructs, ability to minimize drug degradation, reduced toxicity, enhanced permeation through tissues, and higher bioavailability [91,92,93]. A detailed review on types of micelles, methods of preparation and studies involving micelles for both anterior and posterior segment delivery is described by Mitra et al. [94].

#### 3.7.1. Surfactant Nanomicelles

Amphiphilic molecules usually possess a hydrophilic head and a hydrophobic tail. The hydrophilic head group carries either an anionic or cationic charge (ionic surfactant), both positive and negative charges (zwitterion surfactant), or no charge (nonionic surfactant). Sodium dodecyl sulfate (SDS) is an example of anionic surfactant, while dodecyltrimethyl ammonium bromide (DTAB) is an example of cationic surfactant, and dioctanoyl phosphatidylcholine (C8-lecithin) is an example of a zwitterionic surfactant. Non-ionic surfactants, such as dodecyl tetra (ethylene oxide) (C12E4), vitamin E TPGS, and octoxynol-40 are considered to be the least toxic [95,96]. The surfactants that are used at lower concentrations are likely to be absorbed at the surface or the interface, thus lowering the surface or interfacial free energy. Surfactants tend to form clustered aggregates (micelles) at or above the critical micelle concentration (CMC). Micellization occurs when a balance exists between the intermolecular forces, such as hydrophobic, steric, electrostatic, hydrogen bonding, and Vander Waals forces. Other parameters that dictate micellization include the shape and size of surfactant monomers, ionic strength, pH, temperature, total surfactant concentration, and the number of surfactants used [91,92,97]. Several reports published in the literature supported the use of surfactant micelles for improved penetration of topically applied drugs through the cornea and enhanced ocular bioavailability.

Mitra et al. reported a mixed nanomicellar system of vitamin E TPGS and octoxynol-40 for delivery of drugs, such as voclosporin, dexamethasone, and rapamycin. An in vivo study conducted in rabbit eyes and canines revealed enhanced anterior segment drug bioavailability, with no symptoms of ocular irritation or toxicity [94,98]. Vadlapudi et al. solubilized biotin-12hydroxystearic acid-acyclovir (B-12HS-ACV) in nanomicelles of vitamin E TPGS and octoxynol-40 and evaluated their biocompatibility in human corneal epithelial cells (HCECs). B-12HS-ACV released in a sustained manner from the nanomicellar formulation for a period of four days, compared to a 100% release of B-12HS-ACV in 6 h with the ethanol solution [99]. Kuwano et al. compared the pharmacokinetics and distribution of cyclosporine A resulting from its topical application as an oil-based medium (polyoxyl 60 hydrogenated castor oil (HCO-60)), o/w emulsion, and cyclosporine A aqueous clear solution containing a surfactant (polyoxyl 40 stearate (MYS-40)) in rabbit ocular tissues. Higher solubility was reported for the insoluble cyclosporine A with surfactants when compared to the other oil-based medium and emulsion. In vivo studies revealed MYS-40 as a solubilizer of cyclosporine A, showing improved ocular drug accumulation with a single topical administration compared to other formulations. Also, a significant increase in AUC was observed in the corneal stroma-endothelium, bulbar conjunctiva, and lacrimal gland as compared to oil- and emulsion-based formulations [100].

#### 3.7.2. Polymeric Nanomicelles

Polymeric nanomicelles are synthesized from block copolymers. They form amphiphilic monomeric units with distinct hydrophilic and hydrophobic monomeric units that have a hydrophobic core surrounded by the hydrophilic shell. They contain polymer chains that are self-assembled due to hydrophobic or ion pair interactions between polymer segments [94]. The polymer blocks are arranged differently as diblock (A-B type), triblock (A-B-A type) or even grafted or branched type copolymers, where A and B are different polymers. Ideally, the polymers used to prepare nanomicelles should be biodegradable and/or biocompatible [90]. The polymers commonly used in the preparation of nanomicelles include polyethylene glycol, polyethylene oxide, poly (d,l-lactic acid), polypropylene oxide, polyamino acids, such as polyaspartic acid, polyglutamine acid, poly-l-lysine, and poly-histidine [70]. As the length of the hydrophilic segment increases, copolymers tend to exist in aqueous solvents as unimers. Copolymers form non-nanomicellar structures known as rods and lamellae, with an increase in the length of hydrophobic segment [101]. If the core-forming block structures are efficiently monitored, nanomicelles may have good thermodynamic and kinetic stability, enabling a variety of drugs to be incorporated. It was found that polymeric micelles are more stable than nanomicelles made from conventional surfactants [101]. Literature suggests that these polymeric nanomicelles are able to retain drug molecules for a long time, even in a diluted environment in systemic fluids due to their low CMC values [102]. Polymeric nanomicelles have also been employed in active drug targeting [103]. Polymeric micelles offer advantages like extended circulation time, sustained release, favorable biodistribution, and reduced side effects with lower toxicity [92,104,105]. Thus, polymeric nanomicelles are an attractive option for ocular drug delivery.

**Figure 7:**
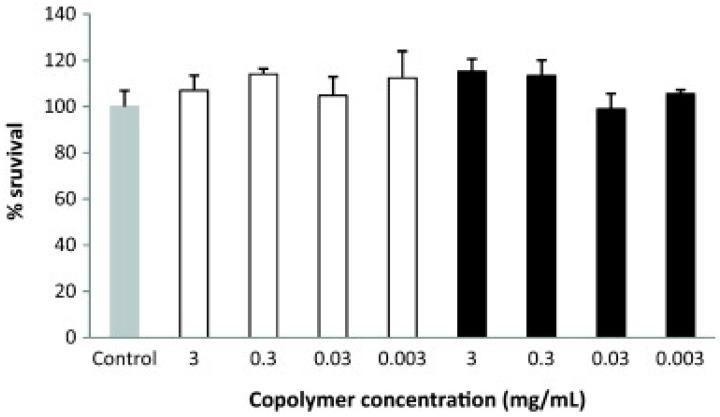
Cell viability of human corneal epithelial cells (HCE) when treated with different concentrations of blank (**white columns**) and cyclosporine A-loaded micelles (**dark columns**). Reprinted from [107]. Copyright (2018), with permission from Elsevier.

Polymeric micelles containing ketorolac and copolymers such as *N*-isopropyl acrylamide (NIPAAM), vinyl pyrrolidone (VP), acrylic acid (AA) cross-linked with *N*, *N*′-methylene bis-acrylamide (MBA) were able to improve the ocular bioavailability two-fold, with no toxicity, when compared to an aqueous suspension containing the same amount of ketorolac [106]. Cyclosporine A was formulated in polymeric micelles of methoxy poly (ethylene glycol)-hexylsubstituted poly (lactides) (MPEG-hexPLA). Results indicated excellent in vitro (Figure 7) and in vivo ocular biocompatibility, transparency, and the stability of the formulation.

These studies demonstrated the potential of MPEG-hexPLA micelles as carriers for cyclosporine A in treating dry eye syndrome, autoimmune uveitis, or for the prevention of corneal graft rejection [107]. Pilocarpine formulated with triblock copolymer Pluronic F127 poly (oxyethylene)/poly (oxypropylene)/poly (oxyethylene) demonstrated improved pharmacokinetics and prolonged miotic response when compared to standard pilocarpine solutions. Such augmentation in mitotic response was attributed to the productive absorption of drug-loaded nanomicelles [108]. Pepic et al. developed and characterized a dexamethasone-loaded nanomicellar formulation with polyoxyethylated nonionic surfactant Pluronic F127 and chitosan. The in vitro release of dexamethasone and transport across Caco-2 cell monolayers was found to be higher in the presence of chitosan compared to chitosan-free Pluronic F127 micelles. Polymeric micelles, Pluronic F127 and chitosan, showed excellent ocular bioavailability with two- to four-fold increases compared to standard dexamethasone suspension. The improved intraocular absorption of dexamethasone from the micellar systems was attributed to the higher permeability and mucoadhesive nature of chitosan [109]. Metipranolol was delivered by using a chitosan-pluronic nanomicellar formulation. Despite the immediate release of the drug with 0.5% chitosan/pluronic micelles, a 1.67-fold increase in area under the curve (AUC) relative to commercial metipranolol eye drops was observed. The elevation in AUC of chitosan/pluronic micelles could be attributed to the bioadhesive nature of chitosan. However, no significant difference in AUC was observed with commercial eye drops of metipranolol when only F127 was used. No change in AUC was attributed to the lack of bioadhesion, leading to the elimination of micelles from the precorneal area [110].

### 3.8. Polyion Complex Nanomicelles

These nanomicelles are formed by electrostatic interactions between polyion copolymers (composed mainly of the neutral segment and ionic segments) and oppositely charged ionic drugs [111]. Polyion complex (PIC) nanomicelles are mainly employed for gene and antisense oligonucleotide delivery [112,113]. In general, the block copolymer is hydrophilic in nature, the neutral block is polyethylene glycol (PEG), and the ionic block is neutralized by oppositely charged species that form the hydrophobic core [114]. For ocular drug delivery, PEG stabilizes the hydrophobic polyion-drug complex, thus forming the PIC micelles. PIC micelles offer reduced side effects as they are target specific and thus are promising carrier systems for ocular delivery of ionic macromolecules [90]. Photodynamic therapy (PDT) with dendrimerporphyrin (DP) loaded PIC micelles was successfully utilized for selective drug accumulation in the pathologic corneal neovascularization area without affecting healthy ocular vessels. Dendrimer zinc porphyrin (DP, photosensitizer) was prepared with PEG-P (Lys) block copolymer, resulting in PIC micelles. PIC micelles accumulated in the corneal neovascular tissue, following intravenous delivery with no accumulation of DP-micelles in normal limbal vessels, suggesting improved drug targeting and enhanced permeability and retention effects [115].

Nanomicelle eye drops have been tested successfully for gene delivery to the anterior segment ocular tissues. This strategy has been used for treating corneal diseases such as corneal neovascularization, dry eye syndrome, corneal scarring, corneal angiogenesis, and inflammation [94]. Liaw et al. have used a nonionic copolymeric system, poly (ethylene oxide)-poly (propylene oxide)-poly (ethylene oxide) (PEO-PPO-PEO), for ocular gene delivery. Stable and efficient delivery of plasmid DNA encapsulated with the LacZ gene was observed in rabbit and mice eyes. Results indicated a promising potential for copolymers in DNA transfer [116]. Another polymeric system composed of PEO-PPO-PEO was developed to deliver genes for cornea-specific promoters (keratin 12 and keratocan). Significant elevation of β-Gal activity occurred (which was considered as a measurement of transgene expression) after administration of six doses of the DNA-encapsulated micellar system. This was attributed to endocytosis and particle size-dependent paracellular transport of polymeric micelles [117]. The polymeric micellar formulation improved m-RNA levels by 2.2-fold, suggesting an increased permeability of nanomicelles in the anterior segment ocular tissues [118].

### 3.9. Nanoparticles

Nanoparticles are colloidal drug carriers with a size ranging from 10 to 1000 nm [70,119]. Drug-loaded nanoparticles with size ranging from 50 to 400 nm are considered versatile for ocular delivery as they have the ability to overcome physiological barriers and to direct the drug to specific cells, either by passive or ligand mediated targeting mechanisms [120]. Nanoparticles used in ophthalmic preparations are made up of lipids, proteins, natural or synthetic polymers, such as albumin, sodium alginate, chitosan, poly (lactide-co-glycolide) (PLGA), polylactic acid (PLA), and polycaprolactone [121]. Nanoparticles have been used to deliver drugs to both the anterior and posterior eye segments. Nanoparticles have several advantages. They: (1) provide less irritation due to small size; (2) provide sustained drug release, thus avoiding frequent administration; (3) prevent premature degradation or non-specific uptake; (4) improve intracellular penetration and provide better absorption; and, (5) provide target-specific delivery to the desired tissue [119,122]. However, they have the tendency to drain from the precorneal pockets, similar to aqueous solutions. Nanoparticle-mediated drug targeting is successfully employed to treat ocular diseases involving angiogenesis, such as central retinal vein occlusion (CNVO), choroidal neovascularisation (CNV), and diabetic retinopathy (DR) [62]. Both lipophilic [123] and hydrophilic drugs [124] can be loaded into nanoparticles. Hydrophilic drugs are entrapped in PLGA nanoparticles using a water-in-oil-in-water (W/O/W) double emulsion technique, whereas hydrophobic drugs are entrapped in PLGA nanoparticles using an oil-in-water (O/W) emulsion technique [119].

Polymeric nanoparticles employ biodegradable and non-biodegradable polymers for sustained drug delivery in the treatment of anterior segment inflammations. To overcome the precorneal elimination, the nanoparticles are often coated with polyethylene glycol, chitosan-hyaluronic acid, and even thermosensitive gels to impart mucoadhesiveness [121]. An extensive review on nanoparticles for anterior segment delivery was published by Dileep et al. [125]. Gan et al. prepared self-assembled liquid crystalline nanoparticles of ethyl rhodamine B (Rh B) employing monoolein and poloxamer 407. The bioavailability of Rh B from nanoparticles increased 3.5 and 2.5-fold when compared to Rh B solution and Rh B carbopol gel, respectively. Further, the precorneal clearance was slower with Rh B nanoparticles as compared to solution and gel formulations [89]. Other drugs, such as ibuprofen, flurbiprofen, and indomethacin were incorporated into nanoparticles for treating anterior segment inflammation. Ibuprofen-loaded nanoparticles were able to improve the bioavailability of the drug in the aqueous humor of rabbit eyes when compared with ibuprofen aqueous eye drops [126]. Similarly, flurbiprofen-loaded in nanoparticles showed improved interactions with the corneal surface with increased drug absorption. This is attributed to the cationic nanoparticles interacting well with the anionic corneal surface, improving the bioavailability [127,128]. Chitosan is the most widely employed polymer for increasing the precorneal residence time of nanoparticles. Cyclosporin A-loaded nanoparticles of chitosan have a positive zeta potential with smaller particle size. These nanoparticles improved the precorneal retention by two-fold in cornea and four-fold in conjunctiva when compared to cyclosporin A eye drop solution or suspensions [129]. Improved ocular bioavailability was observed when gatifloxacin was incorporated into mucoadhesive polymer (HA) coated with Eudragit nanoparticles (RS 100 and RL 100) [130]. Mitra et al. developed a pentablock copolymer system, which is capable of forming nanoparticles with thermosensitive gelling capacity. These polymeric systems have been used to deliver drugs for treating chronic anterior ocular diseases [131]. Human cornea and conjunctival cells have CD44 HA receptors located on them. They were monitored to verify the uptake of hyaluronic acid-chitosan oligomer based nanoparticles (HA-CSO NPs), and it was proven that HA-CSO NPs undergo an active transport mediated with the help of CD44 HA receptors via the caveolin-dependent endocytosis pathway [132]. Active drug targeting using surface-modified nanoparticles has also been pursued by ophthalmic researchers. Nanoparticles functionalized with antibodies, vitamins, peptides, and aptamers showed enhanced uptake in specific ocular tissues. According to Kompella et al. surface functionalized nanoparticles with deslorelin and transferrin showed 64% and 74% higher transport, respectively, when compared to un-functionalized nanoparticles. This indicates that surface modification provides rapid and efficient delivery of nanoparticles into and/or across the cornea and conjunctiva [133]. The efficiency and toxicity of 2 kDa polyethylenimine conjugated to gold nanoparticles (PEI2-GNP) in delivering genes to the human cornea (in vitro) and rabbit corneas (in vivo) was investigated. The hybrid nanoparticles could effectively deliver genes to the human cornea without altering cell viability, while appreciable particle uptake was observed throughout the rabbit stroma, with gradual clearance over time. Further, slit-lamp biomicroscopy performed in live animals following topical administration detected no inflammation or redness, with only moderate cell death and immune reactions, suggesting its potential use in the corneal gene therapy [134]. Nevertheless, the nanoparticles have some disadvantages, such as low drug loading and burst release of drugs. Nanoparticles generally exhibit a biphasic release pattern, with an initial burst release followed by sustained release. Jwala et al. demonstrated that the burst release of drugs from nanoparticles could be eliminated by dispersing nanoparticles in thermosensitive gels such as PLGA-PEG-PLGA [135].

Nanoparticles-loaded into contact lenses are considered as an alternative because they can maximize the residence time of the drug and thus improve the drug permeation through the cornea. They also provide a continuous drug release due to the slow diffusion of drug molecules through nanoparticles and the lens matrix. Drugs can be loaded into the nanoparticles either by soaking the contact lens in a drug solution or by entrapping drugs in nanovesicles and then dispersing these vesicles throughout the contact lens. However, there are a few limitations associated with both methods. Soaked contact lenses deliver drugs only for a limited time period, and the amount of the drug to be loaded depends on the equilibrium solubility of the drug in the lens matrix, which is generally small for most drugs. Thus soaked contact lenses are not a convenient alternative for long-term drug delivery, whereas the entrapment of drugs in nanoparticles results in an additional barrier that prevents the immediate release of drugs [62]. Kim et al. were able to demonstrate that silicon hydrogel material releases timolol and dexamethasone in a zero-order fashion for up to 120 days with a negligible burst release of <5% on the first day [136]. Nakada et al. developed a compound contact lens with a hollow cavity that binds two contact lenses. This, when soaked in the drug solution, resulted in drug loading and allowed the drug to be released following insertion into the eye. However, low oxygen and carbon-dioxide permeability limit its use [137]. Vitamin E-loaded silicone lenses act as barriers to drug diffusion, resulting in an increased duration of action. This approach was investigated using topical anesthetic drugs, including lidocaine, bupivacaine, and tetracaine. Results indicated that vitamin E-loaded lenses could release the anesthetics over seven days, making it highly useful in reducing postoperative pain in patients who undergo corneal procedures such as photorefractive keratectomy [138]. The molecularly imprinted contact lens of ketotifen fumarate was prepared using poly (HEMA-co-AA-co-AM-co-NVP-co-PEG200DMA). The prepared lenses demonstrated greater mean residence time that is four- and 50-fold higher than non-imprinted lenses and commercial eye drops (Zaditor^®^), respectively [139].

### 3.10. Solid-Lipid-Nanoparticles (SLN)

**Figure 8:**
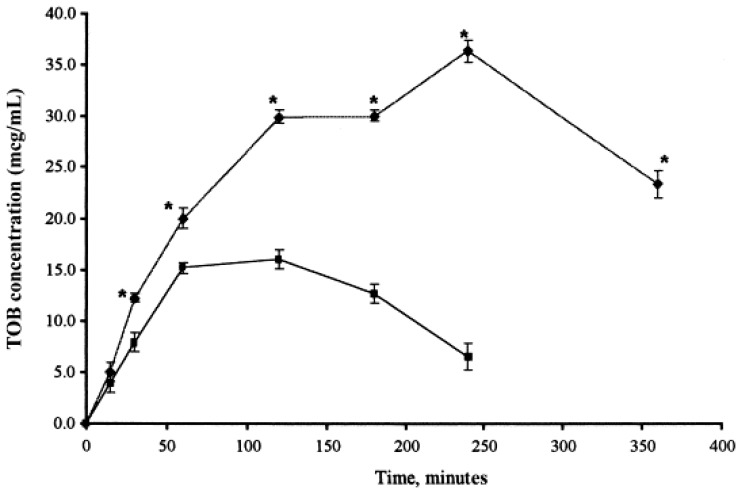
Concentration-time profile of Tobramycin (TOB) in the aqueous humor of rabbit after the instillation of the reference solution (■) and TOB-SLN (♦). Reprinted from [147]. Copyright (2018), with permission from Elsevier.

SLN can be defined as a solid lipid matrix in the nanometer size range accommodating a drug that is stabilized by one or more surfactants [140]. They offer advantages, such as controlled drug release, drug targeting, long-term stability, and biocompatibility due to the use of physiological lipids [141]. However, SLNs have a limited drug-loading capacity (around 25% of lipid matrix) and lead to a burst release of hydrophilic drugs during the initial period [142]. SLNs prevent or reduce the degradation of lipophilic drugs, as the mobility of the reactive agents is hindered in the solid-state compared to the liquid state [143]. Impediments associated with SLN led to the modification of SLN to a nanostructured lipid carrier (NLC). NLCs are capable of accommodating a larger quantity of drugs and hence improve the drug-release profile. The additional space between the fatty acid chains of glycerides allows for more drugs to be accommodated, and the formation of lipid crystals prevents drug expulsion during storage [144]. NLC contains around 30% liquid lipids, but the final product is in solid state with no crystalline structure [140]. SLNs are considered a favorable alternative for ocular drug delivery due to their nano-size range, enhanced corneal absorption, improved bioavailability and prolonged ocular retention in the conjunctival sac. Further, SLNs are easily dispersible in aqueous media and can be formulated into eye drops [145]. Drugs loaded in SLNs are capable of crossing the corneal epithelium, and the anionic nature of the corneal epithelium enables absorption of cationic SLNs [146]. Cavalli et al. demonstrated that SLNs enhance the ocular bioavailability of tobramycin by increasing the residence time on the corneal surface and conjunctiva when compared to an equal dose of tobramycin aqueous solution (Figure 8) [147].

SLNs of diclofenac sodium were developed with a combination of homo-lipid from goat (goat fat) and phospholipid (phospholipon 90 G^®^), and the control formulation contained diclofenac sodium without phospholipid. Drug-loading capacity was approximately 90% and SLNs showed a sustained release of the drug. On the other hand, the control formulation showed low drug loading with a burst release. Permeation across the cornea was improved due to high drug loading and sustained release [148]. Gokce et al. prepared an SLN formulation of cyclosporine A, employing Compritol 888 ATO as the base lipid. The entrapment efficiency of cyclosporine A was found to be 95%. SLNs of cyclosporine A were further tested in rabbit eyes. Results showed that the therapeutic level of cyclosporine A was achieved in aqueous humor after 3 h, which was attributed to internalization/uptake of SLNs by the corneal epithelium. SLNs of cyclosporine A were retained in the precorneal area for up to 40 min, while the retention time of cyclosporine A solution was only 10 min [149].

NLCs due to increased liquid-lipid concentration result in enhanced ocular retention and improved corneal penetration. Shen et al. incorporated cyclosporine A in NLC with 2% Tween 80 as an emulsifier and 2% polyethylene glycol stearate (PEG-SA) as a surface modifier. This resulted in smaller particles with an enhanced drug-loading capacity, although there was no significant difference in entrapment efficiency. The release profile was improved with NLCs, with a faster drug release during the first 12 h, followed by a sustained release, prolonging ocular retention and corneal permeability [150].

### 3.11. Nanoparticle-Laden Devices

To further improve the therapeutic duration and bioavailability, nanoparticles were embedded into a matrix such as hydrogel or hydrogel-contact lenses in order to form a composite drug delivery system. First, the drug needs to diffuse from the nanoparticles to reach the hydrogel/contact lens matrix, and then diffuse from the matrix to the site of action. Therefore, the drug-release duration of the combined system is longer than the release from nanoparticle or hydrogel/contact lens matrix alone [151]. Additionally, the drug metabolism from the enzymes present in tears and on the corneal surface is minimized [44]. Various types of nanocarriers, such as liposomes [152,153,154], lipid-based nanoparticles [155,156], micelles [157,158], polymeric, and metal nanoparticles [44,151] have been investigated for extended release in composite systems. A few studies involving composite systems are described in this current section. Furqan et al. have developed pH sensitive, cyclosporine-loaded Eudragit S100 nanoparticle-laden contact lenses. The nanoparticles were manufactured by Quasi-emulsion solvent diffusion technique using different weights of drug to Eudragit S100. This study showed that the percentage swelling and optical transparency were improved with prepared lenses when compared to control (DL-50, prepared by direct drug entrapment). The optimized lenses had a 1:1 (drug: Eudragit) sustained the drug release over 156 h without affecting the optical and physical properties, whereas in rabbit tear fluids, sustained drug release for up to 14 days was observed. Moreover, no drug leaching was observed in the packaging solution, as confirmed from the packaging study [159].

Another study conducted by Amr et al. investigated the effect of experimental parameters, such as the composition of the polymer mixture and the amount of active ingredient on the preparation of polymeric drug delivery system for prednisolone. Drug-loaded PLGA nanoparticles were prepared by a single-emulsion solvent evaporation technique and were incorporated into the contact lens mixture. The size of the nanoparticles was mostly affected by the amount of co-polymer (PLGA), while the drug loading was affected by the amount of active ingredient used. The prepared lenses were clear, transparent and displayed desired wettability. Nanoparticles alone released 42.3% over 24 h, while nanoparticle-loaded lenses demonstrated a slow release of 10.8%. However, a decrease in hydration by 2% and light transmission by 8% was observed with nanoparticle-loaded lenses when compared to control (unloaded nanoparticle lenses) [160]. The in vitro and in vivo efficacy of timolol maleate (TM)-loaded ethyl cellulose nanoparticle-laden ring in hydrogel contact lenses was recently investigated. The lenses were manufactured by dispersing TM-loaded ethyl cellulose nanoparticles prepared by double-emulsion technique into acrylate hydrogel, which was then fabricated as a ring implant. Later, the same was implanted in hydrogel contact lenses. The study showed that the prepared lenses were non-toxic and non-irritant and showed a sustained drug release for 168 h. Furthermore, in vivo pharmacokinetic data showed a significant increase in the mean residence time (MRT) and area under curve (AUC) with prepared lenses when compared to eye drops. Also, the rabbit model showed a sustained reduction in IOP for 192 h [161].

Liposomal based ion-sensitive in situ gel for the delivery of TM was investigated by Yu et al. Initially, liposomes were produced by the reverse evaporation technique coupled with a pH gradient method (REVPR), and were then incorporated into deacetylated gellan gum gels. The results indicated that TM liposomes were round in shape and demonstrated a 1.93-fold increase in the apparent permeability coefficient when compared to TM eye drops.

**Figure 9:**
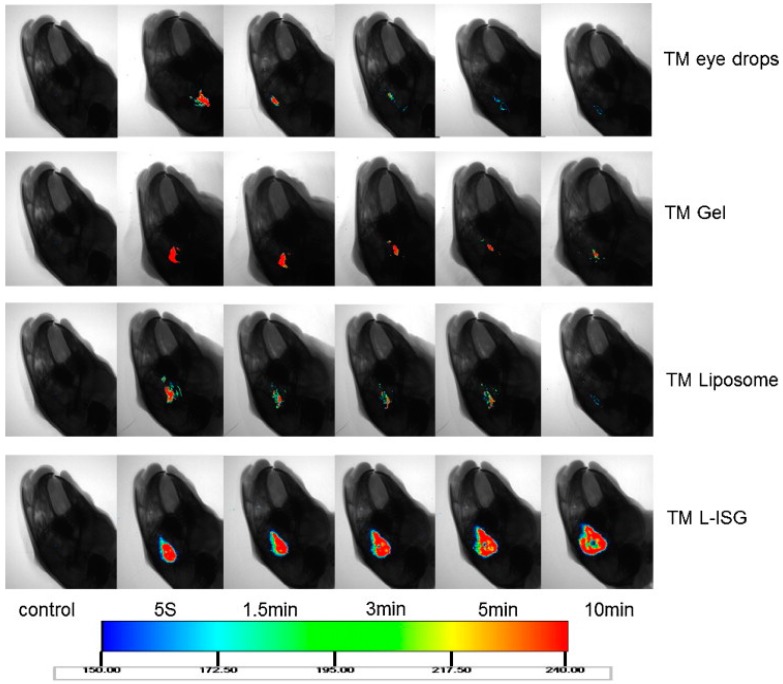
In vivo real time fluroscence imaging of timolol maleate (TM) eye drops, gel, liposomes and composite system (TM L-ISG). Recovery of TM on the corneal surface at 5 s, 1.5, 3, 5 and 10 min post-application. Reprinted from [162]. Copyright (2018), with permission from Elsevier.

On the other hand, the composite system (TM L-ISG) did not show any irritation when tested by draize test and demonstrated a much longer retention time on the corneal surface in vivo when compared to TM eye drops, TM liposomes and TM gel (Figure 9). A quick reduction in IOP was observed when compared to eye drops [162]. A novel in situ gel-based system using Pluronic P123 (P123)/D-α-tocopheryl polyethylene glycosuccinate (TGPS) mixed micelles and gellan gum was investigated for the delivery of curcumin. A central composite design-response surface method was used for optimizing curcumin-loaded P123/TGPS mixed micelles (CUR-MMs). Micelles were initially manufactured, and then were dispersed into 0.2% *w/w* gellan gum solution, which presented a transparent appearance after preparation. The results indicated that the prepared composite system (CUR-MM-ISG) was biocompatible and demonstrated a 1.32-fold increase in cumulative drug permeation compared to curcumin solution [157].

### 3.12. Nanowafers

Nanowafers are tiny circular discs or rectangular membranes containing an array of drug-loaded nanoreservoirs applied to the ocular surface using a fingertip. They release the drug over a longer period of time, thereby increasing the therapeutic efficacy. During the course of drug release, the nanowafers dissolve and fade away [163]. Xiaoyong et al. demonstrated the efficacy of axitinib-loaded nanowafers in treating corneal neovascularization using a murine ocular-burn model. Images from laser scanning microscopy and RT-PCR study results revealed that axitinib-loaded nanowafers administered once a day were twice as effective as axitinib eye drops that were delivered two times a day. Nanowafers were found to be nontoxic and did not affect the wound healing and epithelial recovery processes in the induced model [164].

Nanowafers loaded with dexamethasone (Dex-NW) were developed to improve convenience and efficacy in dry eye patients. The Dex-NW nanowafers were fabricated using carboxymethyl cellulose and composed of 500 nm square-shaped reservoirs filled with dexamethasone. In vivo studies in an experimental mouse model demonstrated that Dex-NW administered once a day alternatively over a five-day treatment period was able to help in the restoration of a healthy ocular surface and corneal barrier function. This effect was comparable to dexamethasone eye drops administered twice daily. Moreover, Dex-NW was able to downregulate the expression of inflammatory cytokines (TNF-α, IFN- chemokines, and MMP-3), which are stimulated by dry eye [165]. A similar study using Dex-NW also reported an effective suppression of corneal inflammation, which was equivalent to the conventional eye drops, even at a four-fold lower concentration and alternative day dosing frequency [166]. Another study conducted by Daniela et al. demonstrated the efficacy of cysteamine (Cys) nanowafers in the treatment of corneal cystinosis. In vivo studies in cystinosin knockout mice revealed that nanowafers containing 10 µg of Cys administered once a day were twice as effective as 44 µg of Cys delivered through topical eye drops. Furthermore, the nanowafers were able to stabilize Cys for up to four months at room temperature when compared to eye drops, which were only stable for up to a week at refrigerated conditions [167].

## 4. Disposition of Nanocarriers Following Topical Application

**Figure 10:**
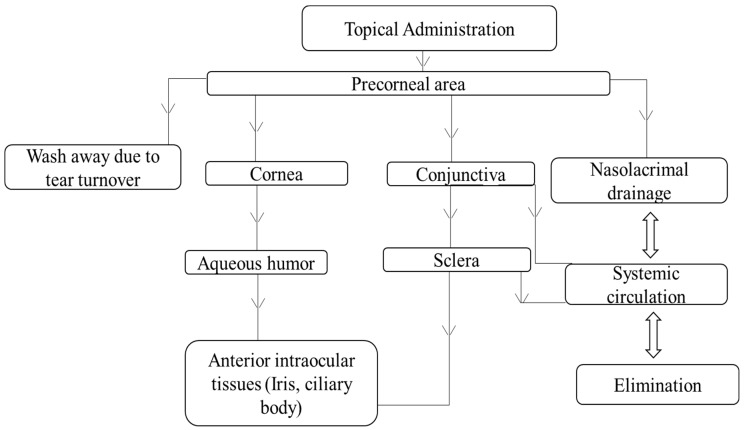
Possible disposition routes of nanocarriers following topical administration.

Following topical application in the form of eye drops, nanocarriers quickly associate with cornea and conjunctiva and are likely disposed via possible routes as shown in Figure 10. However, a major portion of instilled formulation is drained via the nasolacrimal duct. Nanocarriers entering the cornea and conjunctiva predominantly contribute to the drug levels in the anterior segment tissues, while the carriers drained through the nose might be further disposed into gastrointestinal tissues, leading to systemic drug absorption [121]. Ocular disposition of nanocarriers is generally evaluated using various in vitro, ex vivo, and in vivo models. In vitro models used for investigation include human corneal and conjunctival cells and rabbit conjunctival epithelial cells. For ex vivo studies, freshly isolated rabbit cornea and conjunctiva, along with porcine and bovine corneas, were used. Additionally, fluorescent labels including fluorescein, rhodamine, 6-coumarin, and radioactive labels, such as carbon-12 and indium-111 were also used [121].

There are a very limited number of studies on disposition of nanocarriers following topical administration. The transport pathway of poly(n-butyl cyanoacrylate) nanoparticles through the rabbit cornea and conjunctiva was investigated by Zimmer et al. Nanoparticles were prepared by emulsion-polymerization technique and labelled using rhodamine 6 G or propidium iodide. Freshly excised tissues were incubated with a suspension of labelled nanoparticles and were visualized using laser scanning confocal microscopy. Nanoparticles were observed inside the conjunctival tissue, in what appeared to be vesicles or granules. As mentioned by the authors, the possible explanations for this could be either endocytosis or lysis of the cell wall by nanoparticle metabolic degradation products. Also, a florescence signal was observed with corneal cells. Furthermore, a transcellular pathway and penetration only into the first two cell layers were observed [168]. In vivo ocular disposition of poly-hexyl-2-cyanoacrylate nanoparticles in tears, aqueous humor, cornea, and conjunctiva was investigated using radiotracer techniques. The results indicated that a majority of nanoparticles rapidly drained away, while a small percent adhered to the surfaces of the cornea and conjunctiva. Degradation of nanoparticles was observed in tear samples. The levels of nanoparticles in the conjunctiva remained fairly constant, while those in the cornea decreased, slowly, over the time of study. Radioactivity observed in the aqueous humor was attributed to the degradation of nanoparticles rather than endocytosis. Furthermore, increased conjunctival levels of nanoparticles were observed due to the mucolytic agent, *N*-acetyl-l-cysteine. However, no effect of mucolytic agent was seen on the corneal levels of nanoparticles or on the radioactivity observed in the aqueous humor [169].

The mechanism of interaction of rhodamine 6 G-loaded PECL nanocapsules with the corneal and conjunctival epithelia was investigated by Calvo et al. In vitro studies revealed the presence of fluorescent signals only in the epithelial cells, thus demonstrating the intracellular localization of nanocapsules. The mechanism was further confirmed by treating corneas with stained blank nanocapsules, which did not display any fluorescence signals, thus confirming the endocytotic process. In vivo results in rabbits corroborated the uptake mechanism; however, no nanocapsules were noticed in the conjunctival epithelium, indicating a selective interaction of nanocapsules with the ocular tissues [170]. The same group also investigated the ability of different drug carriers, including nanoparticles, nanocapsules, microparticles made of poly-epsilon-caprolactone (PECL) and submicron emulsion, to improve the ocular bioavailability of indomethacin. They observed an increase in the drug concentration in the cornea, aqueous humor, and iris-ciliary body with nanoparticles, nanocapsules, and emulsion, while microparticles hardly showed any effect. Confocal images confirmed that the carriers, which showed improved bioavailability, as penetrated the corneal epithelium by endocytosis. The authors also concluded that similar behavior of these carriers could be due to the specific ingredient in their composition, which either acts as a penetration enhancer or an endocytic stimulator. Also, the colloidal nature of these carriers might be the major factor behind the demonstrated increase in ocular bioavailability [171].

The effect of surface composition on the biodistribution of colloidal drug carriers was investigated by Campos et al. Three types of nanocapsules with varying surface properties were prepared, including poly-ε-caprolactone (PECL) nanocapusles, chitosan-coated PECL nanocapsules, and polyethylene glycol (PEG)-coated PECL nanocapsules. Nanocapsules were loaded with rhodamine dye in order to quantify and visualize their interactions with ocular surface. The results from the ex vivo studies, concluded that the developed nanocapsules, especially the ones coated with chitosan enhanced the corneal penetration of the encapsulated dye. The other conclusion from the confocal laser scanning microscopy is that the nanocapsules were able to enter the corneal epithelium by transcellular route and that the rate of penetration was dependent on the coating composition. The images also revealed that PEG coating accelerated the transport of the nanocapusles across the whole epithelium, while the CS coating favored the retention in the superficial layers of the epithelium. Furthermore, the specific behavior of CS nanocapsules was also corroborated in vivo [172]. In a different study, the characteristics and mechanisms of uptake of PLGA nanoparticles containing 6-coumarin in primary cultured rabbit conjunctival epithelial cells (RCESs) was investigated. The effect of size was studied using three particle sizes (100 nm, 800 nm, and 10 µm). Also, the effect of cytochalasin D, nocodazole and metabolic inhibitors on the uptake of nanoparticles was studied. The maximum uptake at 37 °C occurred at 2 h with 100 nm particles when compared to 800 nm and 10 µm particles. The uptake was confirmed by confocal microscopy and was significantly inhibited by coumarin-free nanoparticles, low incubation temperature, and by the presence of cytochalasin D and metabolic inhibitors. Based on the findings, the authors suggested that the uptake of nanoparticles occurred most likely by adsorptive-type endocytosis [173].

The difference in ocular interactions of fluorescent-labelled chitosan nanoparticles in comparison to solution was investigated. The intensity of fluorescence in the cornea and conjunctiva was evaluated using confocal microscopy and spectrofluorimetry. The results showed that nanoparticles had a greater corneal and conjunctival retention compared to the solution. Also, CS nanoparticles were able to penetrate through the corneal epithelium, as evident from a strong fluorescent signal at the boundary, in addition to a weak signal observed inside the cells. Therefore, suggesting a greater affinity of chitosan in the nanoparticulate form and also a combination of paracellular/transcellular pathways [174]. The delivery of charged nanoparticles in vivo using hydrogel iontophoresis was studied by Eljarrat-Binstock et al. The particle distribution and the penetration efficiency of negatively charged particles compared to positively charged particles into the ocular tissues was also investigated. Strong fluorescene signals were observed in both anterior and posterior ocular tissues. Also, the distribution profile of negatively charged particles revealed a faster uptake into the outer tissues within 30 min of post treatment, followed by migration to inner ocular tissues for up to 12 h. Moreover, positively charged nanoparticles showed better penetration into the inner ocular tissues when compared to negatively charged particles [175].

The effectiveness and mechanism of action of novel nanoparticles made up of two bioadhesive polysaccharides hyaluronic acid (HA) and chitosan for the ocular gene therapy was recently investigated. The nanoparticles were prepared by ionotropic-gelation technique and were loaded with either model plasmid pEGFP or pβ-gal. Transfection and toxicity studies conducted using human corneal epithelial cells (HCE) and normal human conjunctival (IOBA-NHC) cells showed high transfection levels without affecting the cell viability. Furthermore, the confocal images indicated that the nanoparticles were internalized by fluid endocytosis and that the process was mediated by hyaluronan receptor CD44 [176]. In conclusion, more research have to be performed in this area of disposition and transport of nanocarriers. A deeper understanding of disposition and transport mechanisms would therefore contribute towards an efficient anterior segment eye delivery.

## 5. Nanocarriers in Clinical Trials

In spite of the vast research, very few nanocarriers involved in treating anterior segment diseases are now in clinical trials (Table 1). A randomized, single-blind study is currently being conducted to evaluate the efficacy of a microemulsion made of polyunsaturated fatty acids and hydrating polymers (REMOGEN^®^ OMEGA) for treating dry eye (ClinicalTrials.gov Identifier: NCT02908282). The efficacy is being assessed by treating the control subjects with 2% povidone (artificial tears). Recently, Assiut University completed a randomized single-blind phase II trial using urea-loaded nanoparticles for cataract management (ClinicalTrials.gov Identifier: NCT03001466). The efficacy of these nanoparticles has been compared to Balance Salt Solution eye drops (placebo).

In another trial that was conducted by Aston University, the efficacy of different types of artificial tears and a liposomal spray for treating dry eye is being assessed in an interventional randomized study (ClinicalTrials.gov Identifier: NCT02420834). Sun Yat-sen University is also conducting a randomized, single-blind study (ClinicalTrials.gov Identifier: NCT02992392) to compare the efficacy of two tear substitutes (Liposic and Tears Naturale Forte) for dry eye disease. Kala Pharmaceuticals is currently developing mucus-penetrating particles (MPP). This technology is intended to improve drug delivery by enhancing mobility and transport of the particles through the mucus layer. Two product candidates of corticosteroid loteprednol etabonate (KPI-121, 1% and KPI-121, 0.25%) are currently being investigated for their efficacy and safety in treating post-surgical inflammation/pain and dry eye, respectively. The company has completed two phase III trials using KPI-121 (1%) in patients following cataract surgery. The results of these studies revealed that 1% KP1-121 demonstrated statistically significant resolution of both inflammation and pain with twice-daily dosing. Also, it was well tolerated and did not show any adverse effects over the course of trials. Recently, the company filed an NDA application based on the positive results of 1% KPI-121, while 0.25% KP1-121 has successfully completed phase II trial and is currently in phase III trials [177].

The reason for fewer clinical trials may be due to the limitations in the development of nanoformulations. One of the major challenges involved is the toxicity profile of different excipients and polymers employed [178]. Further, a majority of the research on nanoformulations reported in vitro testing and not in vivo. For the in vivo data reported, the rabbit animal model was employed. A rabbit eye does not completely mimic the human eye. Rabbits show higher surface sensitivity, mucus production, and lower blinking rate, resulting in better bioadhesion and ocular retention as compared to human eyes [179].

Further, optimization of complex formulation parameters for various nanocarriers remains a challenge. Drug delivery employing liposomes exhibits limited long-term stability and low drug-loading capacity [180]. With dendrimers, blurring of the vision needs to be addressed [181]. For microemulsions, the formulation stability is affected by the choice of surfactant/co-surfactant and the aqueous/oil phases. Further, higher concentration of the surfactant system demonstrated toxicity [181]. For nanoparticles, aggregation is a major challenge, as it might block the lachrymal drainage punctum, impair the recycling of tear film and contribute to toxicity [182].

## 6. Safety and Toxicity of Nanocarrier Systems

Of critical relevance to the clinical development of nanocarriers intended for ocular use is their ability to be safe and well-tolerated. Only a few studies in the literature detail both in vitro and in vivo toxicological information about nanocarriers. The corneal toxicity of topically applied nanocarriers is studied by histological evaluations and the Draize test. A study by De et al. evaluated two types of brimonidine-loaded polycarboxylic acid nanoparticles for reducing intraocular pressure in glaucoma. An in vitro study using human corneal epithelial cells revealed that polyacrylic acid nanoparticles were biocompatible and non-toxic, while polyitanconic acid nanoparticles were toxic to the cells [183]. Flurbiprofen-loaded PLGA nanoparticles were prepared by Vega et al. In vivo topical instillation of these particles in rabbits showed enhanced anti-inflammatory activity with no signs of irritation or toxicity to the surrounding ocular tissues [184]. Recently, a biopharmaceutical profile of pranoprofen-loaded PLGA nanoparticles dispersed into carbomer hydrogels containing 1% azone was investigated. The study reported no signs of ocular irritancy with hydrogels by both the HEM-CAM in vitro test and an in vivo evaluation in New Zealand rabbits [185]. The interaction of chitosan nanoparticles with ocular mucosa in vivo and also their in vitro toxicity was studied by Campos et al. Confocal microscopy revealed the presence of nanoparticles in the corneal and conjunctival epithelia. Also, the cell survival at 24 h was high, with the viability of the recovered cells being near 100% [186].

Amphotericin B eye drops (Fungizone^®^) showed poor patient compliance due to its potential toxicity. Microemulsion of amphotericin B prepared using a titration technique showed higher anti-fungal activity and better in vitro compatibility with erythrocytes when compared to the eye drop formulation [187]. A similar investigation was done by Ince et al. using pilocarpine microemulsion. The prepared formulation was well tolerated in vivo and showed good stability and reduction in IOP compared to commercial collyrium [188]. A study by Boddeda et al. investigated the efficacy and safety of flurbiprofen nanosuspension in comparision to marketed eye drops. The results from the Draize test and histopathological evaluation revealed that the flurbiprofen-loaded nanosuspension was non-irritant and non-toxic [189]. To address the issue of ocular irritation caused by Restasis^®^, various nanosuspensions loaded with cyclosporine were prepared, and their safety was studied using Draize and Schirmer tests. With the Draize test, both nanosuspension and commercial products caused very slight ocular irritation, resulting in slight redness of the conjunctiva. However, in either case, there was no change in conjunctival discharge when compared to distilled water (control). With the Schirmer test, the nanosuspension showed better results and did not produce any significant differences in flow rates when compared to the commercial formulation [190].

Niosomal formulation of the hydrophilic antibiotic, gentamicin, was studied by Abdelbary and El-Gendy. In vivo studies in albino rabbits over 48 h revealed no signs of redness, irritation, inflammation, or tear production [191]. Another nanocarrier that has been explored recently for ocular delivery is quantum dots. These are semiconductor nanocrystals with unique optical properties. The impact of commonly used CdSe/ZnS core/shell quantum dots (QDs) in the case of corneal abrasion was evaluated by Kuo et al. In this study, QDs at a concentration of 20 nM over 48 h reduced 50% viability in bovine corneal fibroblasts. Also, QDs were found to be retained in the corneal stroma of mice for up to 26 days, suggesting potential cytotoxicity [192].

Thus, taking into consideration, the sensitivity of the eye and the toxicity of nanocarriers and their inherent components, it is of great concern to ensure the safety of ophthalmic nanocarriers before they are made available to the patients. The suitability of nanocarriers with respect to biodegradability, burst/sustained release, and patient comfort for different clinical needs in the anterior segment is yet to be fully explored. Also, attention has to be given to nanocarrier-biological interactions and surface chemistry as they can aid in better understanding of the nanocarrier safety profile.

## 7. Conclusions

Despite tremendous efforts by scientists, eye drops still account for about 90% of the total ophthalmic formulations. This can be attributed to the stringent regulatory requirements for new ocular delivery systems. Promising initial phase clinical trials on nanoformulations report lower doses, less dosing frequency, and high patient tolerance. With recent research advances in nanoformulation development, along with promising in vivo and clinical trial data, nanocarriers have the potential to replace traditional eye drops as a primary choice for topical ocular therapy in the near future.

## Figures and Tables

**Table 1:** Nanocarriers in Clinical trials.

Nanocarriers	Type of Therapy	Stage of Trial	ClinicalTrials.gov Identifier
Microemulsion	Dry eye	Randomized, single-blind study	NCT02908282
Nanoparticles	Cataract management	Randomized single-blind phase II trial	NCT03001466
Different classes of artificial tears including a liposomal spray	Dry eye	Interventional randomized study	NCT02420834
Liposomes	Dry eye	Randomized, single-blind study	NCT02992392
Mucus-penetrating particles (MPP)—Loteprednol etabonate ophthalmic suspension: (a) KPI-121, 1%	KPI-121, 1%—Post-surgical inflammation/pain	KPI-121, 1%—completed phase III trial	NCT02163824
	KPI-121, 0.25%—Dry eye	KPI-121, 0.25%—phase III trial	NCT02813265
(b) KPI-121, 0.25%	-	-	-
